# Supplementary figures and images for: Standards-based audit to improve quality of maternal and newborn care—A stepped-wedge cluster randomised trial in Malawi
Source: PLoS One. 2024 Sep 30;19(9):e0310896. doi: 10.1371/journal.pone.0310896 (PMC11441693; doi:10.1371/journal.pone.0310896)

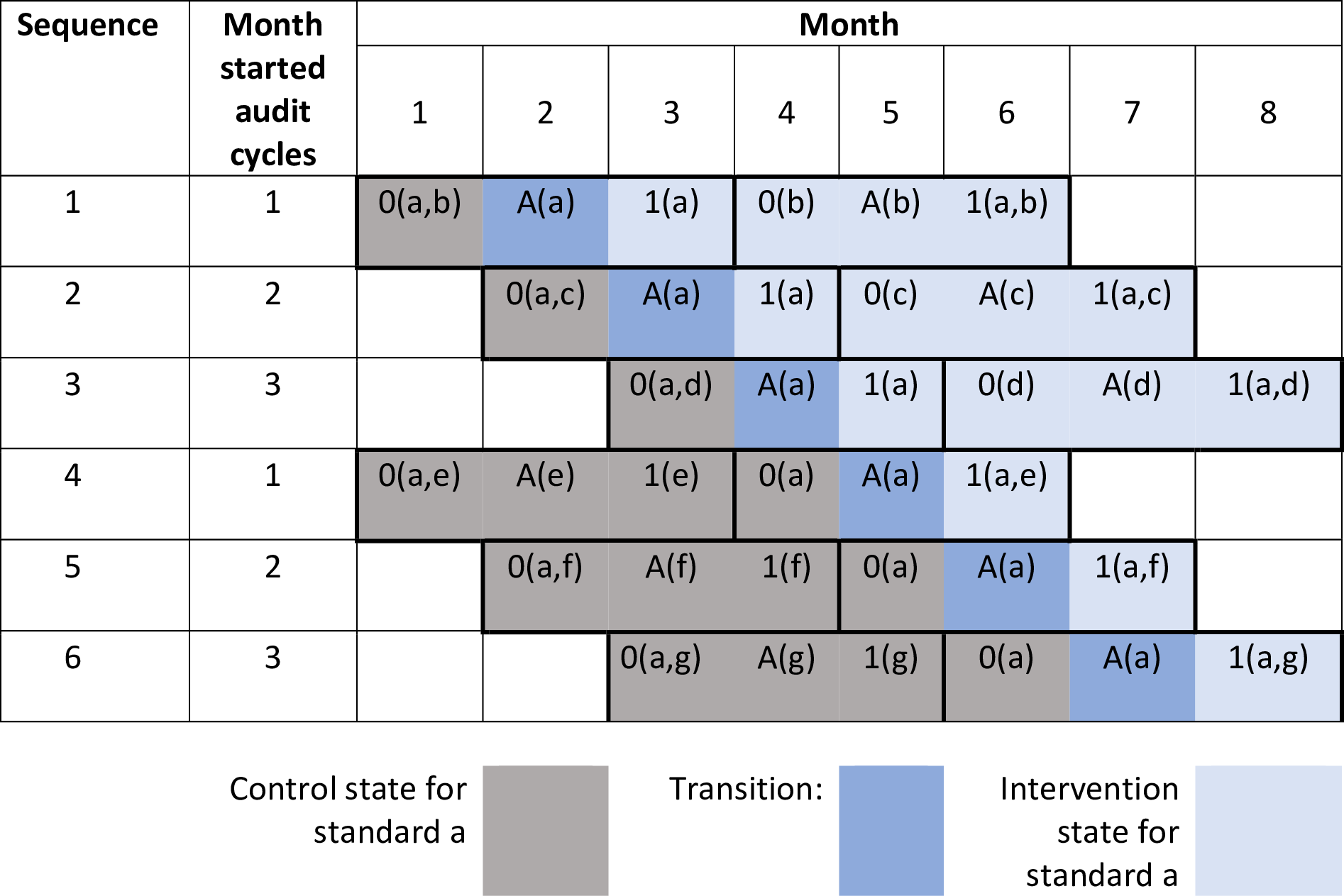

Supplement: S1 Fig — 0() denotes assessment of compliance with the Standard(s) listed under the current standard of care. A() denotes action taken for standard listed, after review of data collected previously. 1() denotes assessment of compliance after taking action to improve the quality-of-care delivery for the standard(s) listed. Standards are indicated by letters a, b… g. Standard a is distinct from standards b.. g. Standards b.. g may all be distinct but were not required to be distinct. When a sequence was used for multiple facilities, the standard audited in the alternative period was not required to be consistent, though a single letter is used to denote it within this figure. Within each sequence the audit cycle periods are indicated by boldly bordered sets of three-month periods. (TIF) [file pone.0310896.s001.tif]

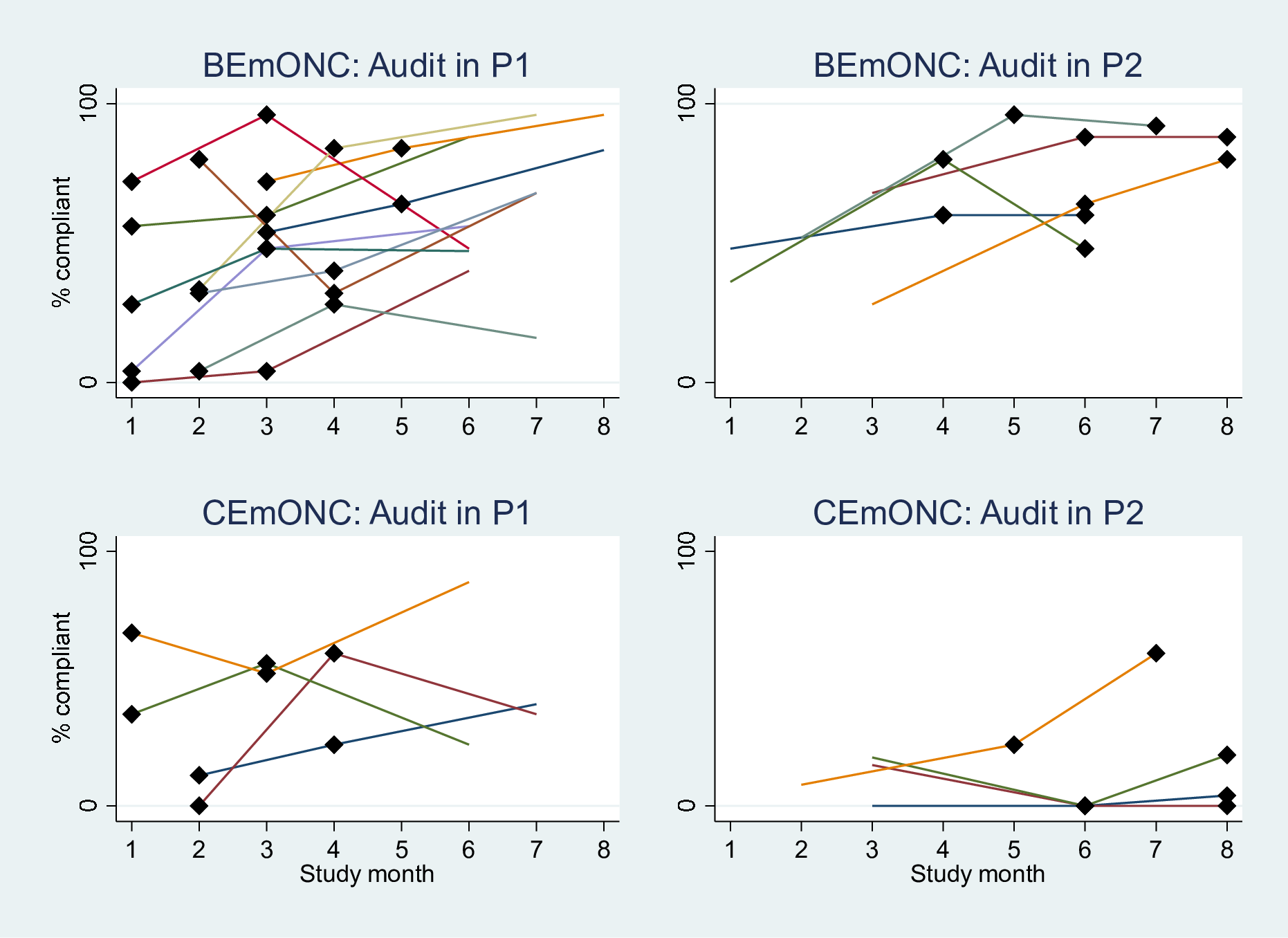

Supplement: S2 Fig — P1 = period 1; P2 = period 2. (TIF) [file pone.0310896.s002.tif]
