# Supplementary material for: Standards-based audit to improve quality of maternal and newborn care—A stepped-wedge cluster randomised trial in Malawi
Source: PLoS One. 2024 Sep 30;19(9):e0310896. doi: 10.1371/journal.pone.0310896 (PMC11441693; doi:10.1371/journal.pone.0310896)
Supplement: S1 Table — (DOCX) [file pone.0310896.s003.docx]

#### S1 Table. Essential components (signal functions) of Comprehensive and Basic Emergency Obstetric and Newborn Care (CEmONC and BEmONC).

| **Signal functions** | **Comprehensive EmONC** | **Basic EmONC** |
| --- | --- | --- |
| 1. Administer parenteral antibiotics (iv or im) | X | X |
| 1. Administer uterotonic drugs (iv or im) | X | X |
| 1. Administer parenteral anticonvulsants for pre-eclampsia and eclampsia (magnesium sulphate) (iv or im) | X | X |
| 1. Manual removal of retained placenta | X | X |
| 1. Removal of retained products of conception by manual vacuum aspiration (MVA) or dilatation and curettage (D&C)^a^ | X | X |
| 1. Assisted vaginal delivery by vacuum extraction/ventouse delivery or obstetric forceps ^b^ | X | X |
| 1. Neonatal resuscitation (with bag and mask) | X | X |
| 1. Blood Transfusion | X |  |
| 1. Caesarean Section | X |  |

Manual vacuum aspiration is preferred.

1. Vacuum extraction/ventouse delivery is preferred.
