# Supplementary material for: Standards-based audit to improve quality of maternal and newborn care—A stepped-wedge cluster randomised trial in Malawi
Source: PLoS One. 2024 Sep 30;19(9):e0310896. doi: 10.1371/journal.pone.0310896 (PMC11441693; doi:10.1371/journal.pone.0310896)
Supplement: S2 Table — (DOCX) [file pone.0310896.s004.docx]

#### **S2 Table. Participating healthcare facilities by district and level of service provision showing strata to which randomised**.

| **District** | **Level of service provision – Basic (B) or Comprehensive (C) Emergency Obstetric and Newborn Care (EmONC)** | **Stratum assigned to in trial** | | |
| --- | --- | --- | --- | --- |
|  |  | I | II | III |
| **Blantyre** | BEmONC | Chilomoni  Lundu  Zingwangwa | Limbe  South Lunzu | Bangwe  Mdeka |
|  | CEmONC |  | Mlambe | Ndirande |
| **Dedza** | BEmONC | Chitowo  Lobi  Mayani | Mtakataka  Mtendere | Chikuse  Chimoto  Golomoti |
|  | CEmONC | Mua | Dedza |  |
| **Mangochi** | BEmONC | Lungwena  Namwera  Phiri Longwe | Chilipa  Chjlonga  Mkumba | Makanjira  Nankumba |
|  | CEmONC | Monkey Bay |  | Koche  Mangochi |
| **Nkhata Bay** | BEmONC | Chintheche  Mzenga | Liuzi  Mpamba | Bula |
|  | CEmONC |  |  | Nkhata Bay |
| **Thyolo** | BEmONC | Khonjeni  Thekerani | Chimaliro  Mikolongwe | Bvumbwe  Chisoka |
|  | CEmONC |  | Thyolo | Malamulo |
