# Supplementary material for: Standards-based audit to improve quality of maternal and newborn care—A stepped-wedge cluster randomised trial in Malawi
Source: PLoS One. 2024 Sep 30;19(9):e0310896. doi: 10.1371/journal.pone.0310896 (PMC11441693; doi:10.1371/journal.pone.0310896)
Supplement: S3 Table — (DOCX) [file pone.0310896.s005.docx]

#### S3 Table. Standards of Care for Emergency Obstetric and Newborn Care (EmONC) developed through consensus by multidisciplinary group of healthcare providers, managers, and researchers.^a^

| General Standards of Care |
| --- |
| - Every woman seeking care for a complication during or after pregnancy is attended to by a skilled healthcare provider within 30 minutes after arrival at the healthcare facility |
| - All women who give birth at a healthcare facility are given the opportunity to have a companion of choice |
| - **All women attending for birth are received and treated with respect (respectful care)** |
| Management of Haemorrhage |
| - Every woman with a retained placenta undergoes manual removal of the placenta within one hour of the diagnosis having been made |
| - **As part of active management of the third stage of labour, all women giving birth at the healthcare facility receive an oxytocic (oxytocic)** |
| - All women who have an antepartum (APH) or postpartum haemorrhage (PPH) have an IV line inserted within 15 minutes of the diagnosis |
| - **All women who have an antepartum (APH) or postpartum- haemorrhage (PPH) have their haemoglobin (Hb) checked and recorded (Hb after APH / PPH)** |
| Management of Infection and Sepsis |
| - Women with a suspected diagnosis of infection or sepsis are started on antibiotic treatment within one hour of the diagnosis having been made |
| - All women who require caesarean section are given prophylactic antibiotics |
| - **All women who have an uncomplicated birth at the healthcare facility have their temperature measured and recorded at least once after birth and before discharge (sepsis detection)** |
| - **All women with fever are tested for malaria within 24 hours (malaria detection)** |
| Management of (Pre-) Eclampsia |
| - Women with high blood pressure during labour or birth are given anti-hypertensive treatment |
| - Every woman who has an eclamptic fit is given magnesium sulphate or diazepam IV or IM within 5 minutes of the fit occurring |
| - **Every woman in labour has her blood pressure measured, urine tested for protein and the results recorded (identification of pre-eclampsia)** |
| - **Every woman with pre-eclampsia or eclampsia has a fluid input-output chart completed (management of pre-eclampsia)** |
| - **All mothers attending ANC have their blood pressure checked and urine tested for protein (ANC pre-eclampsia screening)** |
| **Management of Prolonged and Obstructed Labour** |
| - **Every woman in labour in a healthcare facility is monitored using a partograph correctly (partograph)** |
| - **All women who need an emergency Caesarean Section should be delivered within 60 minutes of the decision (CS timing)** |
| Management of Abortion |
| - Every woman who has had uterine evacuation or MVA has her temperature measured and recorded before she is discharged home |
| - **Every woman with an incomplete miscarriage/abortion undergoes evacuation / manual vacuum aspiration (MVA) within 24 hours of arrival at the healthcare facility (MVA timing)** |
| - Every woman who had an abortion/ miscarriage (complete or incomplete) is provided with advice on contraception before discharge home |
| - **Every woman who has had uterine evacuation or manual vacuum aspiration (MVA) has a clinical examination before being discharged home (MVA exam)** |
| Newborn Care |
| - Every newborn baby delivered in the facility is weighed after birth |
| - All newborn babies with temperature of 38^0^ C or more should be started on antibiotics within 12 hours of the measurement |
| - Low Birth Weight (LBW) babies receive Kangaroo Mother Care (KMC) |

a Standards listed in bold were subjected to audit. The standard name allocated is shown in brackets. These standards were extracted from reference 9.
