# Supplementary material for: Standards-based audit to improve quality of maternal and newborn care—A stepped-wedge cluster randomised trial in Malawi
Source: PLoS One. 2024 Sep 30;19(9):e0310896. doi: 10.1371/journal.pone.0310896 (PMC11441693; doi:10.1371/journal.pone.0310896)
Supplement: S4 Table — (DOCX) [file pone.0310896.s006.docx]

#### S4 Table 4. Numbers of HCFs to which each standard was assigned for audit, and at which audit was completed, by stratum and by period.

| Standard | | Number of HCFs Assigned | | | | | | | | | | | | |  | Number of HCFs which completed audit | | | | | | | | | | | |
| --- | --- | --- | --- | --- | --- | --- | --- | --- | --- | --- | --- | --- | --- | --- | --- | --- | --- | --- | --- | --- | --- | --- | --- | --- | --- | --- | --- |
|  |  | Stratum | | | | | | |  | Period | | | | |  | Stratum | | | | | | |  | Period | | | |
|  |  | I | | II | | III | | |  | 1 | | 2 | | |  | I | | II | | III | | |  | 1 | | 2 | |
| 1 | 6 | | 4 | | 6 | |  | 7 | | | 9 | |  | 4 | | | 4 | | 9 | |  | 6 | | | 11 | |  |
| 2 | 1 | | 1 | | 7 | |  | 3 | | | 6 | |  | 1 | | | 0 | | 8 | |  | 6 | | | 3 | |  |
| 3 | 1 | | 2 | | 0 | |  | 2 | | | 1 | |  | 0 | | | 1 | | 0 | |  | 0 | | | 1 | |  |
| 4 | 6 | | 6 | | 6 | |  | 9 | | | 9 | |  | 7 | | | 6 | | 6 | |  | 12 | | | 7 | |  |
| 5 | 6 | | 5 | | 6 | |  | 9 | | | 8 | |  | 4 | | | 5 | | 5 | |  | 6 | | | 8^a^ | |  |
| 6 | 2 | | 2 | | 1 | |  | 3 | | | 2 | |  | 1 | | | 0 | | 1 | |  | 2 | | | 0 | |  |
| 7 | 0 | | 1 | | 0 | |  | 1 | | | 0 | |  | 0 | | | 1 | | 0 | |  | 1 | | | 0 | |  |
| 8 | 2 | | 2 | | 2 | |  | 4 | | | 2 | |  | 0 | | | 2 | | 1 | |  | 2 | | | 1^b,c^ | |  |
| 9 | 6 | | 6 | | 6 | |  | 9 | | | 9 | |  | 9 | | | 8 | | 7 | |  | 15 | | | 9 | |  |
| 10 | 1 | | 0 | | 3 | |  | 2 | | | 2 | |  | 1 | | | 0 | | 3 | |  | 3 | | | 1 | |  |
| 11 | 1 | | 0 | | 2 | |  | 1 | | | 2 | |  | 1 | | | 1 | | 1 | |  | 1 | | | 2 | |  |
| 12 | 2 | | 3 | | 1 | |  | 2 | | | 4 | |  | 2 | | | 3 | | 2 | |  | 2 | | | 5 | |  |
| **Total** | | 34 | | 32 | | 40 | | |  | 52 | | 54 | | |  | 30 | | 31 | | 43 | | |  | 56 | | 48 | |

a F18 attempted to audit the standard but there were no cases in M1 or M4, so it is not counted

b F19 collected data for the standard but as clients are required to pay at this facility for the service it was not used, so it is not counted

c F28 attempted to audit the standard but there were no cases in M1 or M4 so data for M6 was not used, so it is not counted

HCF = healthcare facility
