# Supplementary material for: Standards-based audit to improve quality of maternal and newborn care—A stepped-wedge cluster randomised trial in Malawi
Source: PLoS One. 2024 Sep 30;19(9):e0310896. doi: 10.1371/journal.pone.0310896 (PMC11441693; doi:10.1371/journal.pone.0310896)
Supplement: S5 Table — (DOCX) [file pone.0310896.s007.docx]

#### S5 Table. Summary of deviations, in sequence and period when Standards were audited, from randomised standards, by facility.

| **Facility** | **Cycle** | **Assigned^a^** | **Audited** | **Deviation(s)^b^ and missing data** |
| --- | --- | --- | --- | --- |
| **Stratum I** |  |  |  |  |
| F2 | 1 | 5 | 9 | S9 more urgent |
| F4 | both | 5 / 9 | 9 / 5 | Sequence reversed; no data collected for S5 in M1 as no means to capture required data |
| F8 | both | X / 1 | 1 / 9 | Trained late, with stratum III; advised to do only cycle 2 of stratum I; chose instead to follow timings for stratum III, auditing S1 and then additionally S9 |
| F10 | 1 | 1 | 9 | S9 more pertinent |
| F12 | 2 | 6 | 9 | S9 in place of S6 as lack of reagents |
| F19 | both | 8 / 10 | 10 / 8 | Sequence reversed for 2 of 4 standards due to lack of reagents for S8; data for S8 not used as Standard not applicable at facility |
| F24 | both | 3 / 9 | 9 / 4 | S3 dropped as not applicable; S9 brought forward and S4 added |
| F26 | 2 | 6 | 6 | No cases in M1 for S6 |
| F27 | Both | 1 / 4 | 4 / 1 | Sequence reversed due to lack of enumerator in cycle 1 for S1 |
| F28 | 2 | 8 | 8 | Only 1 and 5 cases were observed in M4 and M6 when more were available, so the standard was omitted from analysis |
| **Stratum II** |  |  |  |  |
| F5 | 2 | 6 | 11 | S11 instead of S6 |
| F7 | 1 | 6 | 9 | S9 in place of S6 due to lack of reagents |
| F14 | 1 | 8 | 8 | No cases in M6 |
| F16 | both | 1 / 9 | 9 / 1 | Sequence reversed due to some initial lack of clarity regarding S1 |
| F17 |  | 5 / 9 | 5 / 9 | Attended training with stratum I but adhered to assigned schedule (stratum II) |
| F18 | both | 3 / 5 | 9 / 5 | S3 not done due to lack of cases; replaced by S9; S5 attempted but no cases to assess in M1 or M4 |
| F22 | 2 | 6 | 6 | No cases in M2 or M7 |
| F30 | both | 5 / 4 | 4 / 5 | Sequence reversed due to lack of cases for S5 in M1 |
| F31 | 1 | 4 | 4 | Some data for M3 collected in M2 |
| F35 | both | X / 2 | 2 | Trained late; advised to do only cycle 2 in assigned stratum; chose instead to follow timings for cycle 1 in stratum III |
| **Stratum III** |  |  |  |  |
| F20 | both | 8 & 9 /  2 & 4 | 1 & 2 / 4 & 9 | S2 and S9 were done in reverse sequence;  S8 was dropped due to lack or reagents and replaced by S1; M3 data for S1 were collected in M4 and M4 data for S4 were collected in M3 |
| F21 | 2 | 11 | 9 | S11 was replaced by S9 as S9 was perceived to be a bigger problem than S11 |
| F35 |  |  |  | Trained late; advised to do only cycle 2 in assigned stratum; chose instead to follow timings for stratum III for the standard assigned |
| F37 | both | 1/9 | 1/12 | S12 replaced of S9 as S9 was considered good;  data for S1 for M6 collected in M7 |
| F38 | 2 | 6 | 6 | No cases in M3 |
| F39 | both | / 2 & 5 | 1 & 2/ | All 4 standards audited in the first cycle; S5 was dropped due to lack of cases and replaced by S1, one standard assessed in M6 |
| F41 | 2 | 1 | 1 | Some data for M6 collected in M7 |
| F43 | 1 | 6 | 6 | No cases in M3 |

a x / y indicates that Standard x was assigned to be audited in the first cycle and y in the second cycle.

b M1 refers to month 1 of the study for the facility (July in stratum I, August in stratum II and September in stratum III
