# Supplementary material for: Standards-based audit to improve quality of maternal and newborn care—A stepped-wedge cluster randomised trial in Malawi
Source: PLoS One. 2024 Sep 30;19(9):e0310896. doi: 10.1371/journal.pone.0310896 (PMC11441693; doi:10.1371/journal.pone.0310896)
Supplement: S6 Table — (DOCX) [file pone.0310896.s008.docx]

#### S6 Table. Estimates of compliance OR between study arms and correlations for each standard.

| **Standard** | **N** | **Adjusted^a^ OR**  **(95% CI)** | **ICC (HCF)**  **(95% CI)** | **ICC (HCF * month)**  **(95% CI)** | **CAC** |
| --- | --- | --- | --- | --- | --- |
|  |  |  |  |  |  |
| **1** | 1,245 | 19.6  (3.10, 124) | 0.05  (0.002,0.63) | 0.53  (0.40,0.66) | 0.10 |
| **4** | 1,358 | 7.46  (1.79,31.2) | 0.37  (0.19,0.58) | 0.59  (0.44,0.73) | 0.62 |
| **5** | 558 | 6.79  (1.34, 34.3) | 0.48  (0.11,0.86) | 0.50  (0.17,0.83) | 0.95 |
| **8** | 115^b^ | 17.7  (0.86.362) | <0.001  (.,1) | 0.23  (0.02,0.80) | <0.001 |
| **9** | 1,724 | 1.17  (0.48,2.84) | 0.24  (0.13,0.41) | 0.37  (0.26,0.50) | 0.65 |
| **10** | 219 | 1.10  (0.25,4.90) | <0.01  (>0.0001,1.00) | 0.13  (0.04,0.35) | <0.001 |
| **12** | 396 | 2.77  (0.57,13.6) | 0.01  (<0.001,1) | 0.16  (0.06,0.33) | 0.005 |

a adjusted for facility type and month, with random effects for facility and facility-by-month interaction

b 11 values excluded from analysis due to model fitting constraints

HCF = healthcare facility
