# Supplementary material for: Standards-based audit to improve quality of maternal and newborn care—A stepped-wedge cluster randomised trial in Malawi
Source: PLoS One. 2024 Sep 30;19(9):e0310896. doi: 10.1371/journal.pone.0310896 (PMC11441693; doi:10.1371/journal.pone.0310896)
Supplement: S7 Table — (DOCX) [file pone.0310896.s009.docx]

#### S7 Table. Underlying secular trends for Partograph and Sepsis Detection Standards and the aggregate analysis

| **Standard** | **Contrast (95% CI) with Month 1** | | | | | | |
| --- | --- | --- | --- | --- | --- | --- | --- |
|  | **2** | **3** | **4** | **5** | **6** | **7** | **8** |
| **Partograph** | 0.86  (0.24,3.07) | 1.99  (0.66,5.95) | 2.95  (0.75,11.6) | 3.77  (0.76,18.7) | 2.03  (0.63,6.52) | 5.20  (1.06,25.5) | 4.39  (0.71,27.2) |
| **Sepsis detection** | 1.45 (0.17,12.1) | 0.45 (0.07,2.88) | 0.97 (0.12,7.71) | 0.15  (0.01,2.03) | 0.64  (0.09,4.67) | 0.62  (0.04,8.97) | 0.28  (0.02,4.93) |
| **Aggregate** | 1.34  (0.44,4.06) | 1.47  (0.58,3.71) | 1.87  (0.72,4.87) | 1.70  (0.60,4.81) | 2.31  (0.89,5.95) | 2.10  (0.60,7.43) | 1.22  (0.35,4.24) |
