# Supplementary material for: Standards-based audit to improve quality of maternal and newborn care—A stepped-wedge cluster randomised trial in Malawi
Source: PLoS One. 2024 Sep 30;19(9):e0310896. doi: 10.1371/journal.pone.0310896 (PMC11441693; doi:10.1371/journal.pone.0310896)
Supplement: S1 File — (DOCX) [file pone.0310896.s010.docx]

**Using Standards-based Audit to improve Maternal and New-born Health in Malawi - Study Protocol**

**Background**

The latest estimates on mortality show that almost 303,000 women die each year of pregnancy-related causes worldwide and that 99% of these occur in low and middle-income countries^1^. Furthermore, almost three million stillbirths and an estimated 2.6 million neonatal deaths occur per year, the latter accounting for at least 46% of deaths in children aged less than five years^2^. Most of these deaths could be prevented or avoided through actions that are proven to be effective and affordable.

Audits are proven to be effective to improve professional practice^3^ and recently, such audits have been given attention in the domain of obstetric care in low- and middle-income countries^4,5^ . Standard based audit (SBA) also known as criteria-based audit (CBA) is one a useful method which has been used to define and improve quality of care in maternal and newborn health among others (maternal and perinatal death audit and near miss audit)^6,7^. SBA is defined as quality improvement (QI) process that seeks to improve patient care and outcomes by systematically reviewing care against explicit standards, with identification and implementation of changes needed to achieve the desired standard of care^8^. The aim in conducting SBA- is to improve adherence to agreed standards.  If facilities maintain the practices adopted within the audit cycle it is anticipated that care will improve the clients of the facilities will benefit**.**

Recently the World health organisation (WHO) has developed standards for maternal and neonatal health as part of the WHO integrated management of pregnancy and childbirth care (IMPAC) package, which provides guidance for assisting countries to improve the health and survival of women and their newborn babies during pregnancy, childbirth, and the postnatal period^9^. WHO recommends countries to adapt the standards and streamline within national quality of care strategies and frameworks for the delivery of maternal and newborn health services to ensure that the services provided are of high quality.

A complete SBA cycle comprised of five steps: (i) establish a standard, (ii) measure practice, (iii) feedback findings and set standards, (iv) implement change, (v) re-evaluate practice and feedback findings. SBA provides an important insight into deficiencies in clinical practice and are routinely used as part of quality assurance efforts in industrialized countries but underutilized in low- and middle-income countries^10^. Reports of successfully completed cycles on the quality of delivery care are very scanty,

Malawi has achieved significant progress during the past decades in reducing maternal, newborn and child mortality. Child mortality was estimated at 64 per 1,000 live births in 2015, a remarkable progress from the levels registered in 1990 (250 per 1,000 live births). Despite this progress, 15,000 newborns still die in Malawi every year^11^. Maternal mortality was estimated at 957/100,000 live births in 1990 and at 634/100,000 live births in 2015^i^. Maternal deaths represent about 15% of all deaths of women in the reproductive age group (15-49 years), which translates to an estimated 3,400 pregnant women dying every year. With the transition from Millennium Development Goals to the new global Sustainable Development Goals, absolute targets have been set to reduce maternal deaths, stillbirth, and neonatal mortality^12^ and there is still more to be done. To maintain the gains made, and to achieve the global health targets for ending preventable maternal and newborn deaths^13,14^ , a new emphasis on improving quality of care at the time of birth, and for sick newborns, is required in Malawi.

The aim of this study is to assess the impact of using standards-based audit on compliance with defined standards for emergency obstetric and newborn care in Malawi

Study hypothesis:

In Malawi, SBA has been implemented before^4^, however, there is paucity in data to assess how health care providers comply to implement standards selected. Thus, our hypothesis is “Introducing the practice of conducting standards-based audits within facilities providing emergency obstetric and newborn care in Malawi will improve compliance to standards of care”.

# **Methods**

Study design:

A Cluster randomised incomplete stepped wedge trial for multiple standards on obstetric Care will be applied to assess the impact of using standards-based audit on compliance with defined standards for emergency obstetric and newborn care in Malawi. To appropriately reflect the diversity of expert opinion and disciplinary perspectives, a set of standards around emergency obstetric and newborn care were developed using a systematic, participatory process in accordance with WHO process of developing guidelines^15^ . The standards were mainly developed to address the common obstetric and newborn causes of death in low resource settings (cite). There are 25 standards in 7 groups (subsequently referred to as Standards Groups A, B, C, D, E, F and G) which have been defined (Appendix 1)

A total of 44 facilities are included in the design comprising of 10 designated to provide Comprehensive emergency obstetric care CEmOC and 34 facilities designated to provide basic emergency obstetric care (BEmOC) health facilities. Each participating facility is a cluster, and each facility will implement two periods of standards-based audit cycles. Each audit cycle comprises three months (a quarter): an assessment month, a month for action to address issues and a re-assessment month. Each of the ten CEmOC facilities will complete two standards-based audits in each quarter (four standards in total) and each of the 34 BEmOC facilities will complete one standards-based audits in each quarter (two standards in total).

Each standard assessed will be assessed using the incomplete stepped wedge design shown in Figure 1. Up to three facilities will be assigned to each sequence.

**Figure 1: Schematic representation of measurements of compliance with a single standard by sequence and month of study**

| Sequence | Stratum | Month | | | | | | | |
| --- | --- | --- | --- | --- | --- | --- | --- | --- | --- |
|  |  | July | Aug | Sept | Oct | Nov | Dec | Jan | Feb |
| 1 | I | 0 |  | 1 |  |  | 1 |  |  |
| 2 | II |  | 0 |  | 1 |  |  | 1 |  |
| 3 | III |  |  | 0 |  | 1 |  |  | 1 |
| 4 | I | 0 |  |  | 0 |  | 1 |  |  |
| 5 | II |  | 0 |  |  | 0 |  | 1 |  |
| 6 | III |  |  | 0 |  |  | 0 |  | 1 |

0 denotes assessment of compliance with the standard under the current standard of care.

1 denotes assessment of compliance after taking action to improve the quality-of-care delivery for the standard

**Condition**

Recipient of obstetric or new-born care.

**Intervention**

The intervention is the adoption by a healthcare facility of standards-based audits for standards of emergency obstetric and newborn care. Using the stepped wedge design each participating health care facility (cluster) acts as their own control, providing data for standards audited within the facility both prior to and subsequent to the action phase of the audit cycle.

**Trial setting**

Health care facilities

# **Study sites**

A total of 10 facilities designated to provide CEmOC and 34 facilities designated to provide BEmOC, from five districts in Malawi are to participate in the trial. The Table 1 below shows their distribution across the districts. Names of all facilities are in Appendix 2

**Table 1: Distribution of health facilities across the districts.**

| **District** | **CEmOCS** | **BEmOCs** |
| --- | --- | --- |
| Dedza | 2 | 8 |
| Blantyre | 2 | 7 |
| Thyolo | 2 | 6 |
| Mangochi | 2 | 9 |
| Nkhata Bay | 2 | 4 |
| **Total** | **10** | **34** |

**Primary outcome measure**

Compliance with defined standard of care aggregated for all emergency obstetric and newborn care standards audited. This will be defined as the mean across all facilities and standards, with each standard audited by each facility carrying equal weight. (Thus, at facility level the means for CEmOC facilities which audit twice as many standards will carry twice the weight of the BEmOC facilities). Standards will be weighted by the number of facilities which audit the standard).

**Secondary outcome measure**

Compliance with defined standard of care for each of the emergency obstetric and newborn care standards audited in the study.

**Randomisation**

Facilities have been randomised to three strata of 14 or 15 facilities, with each stratum including 2 or 3 CEmOC facilities. The stratum assigned determines the month in which the first audit cycle will be commenced (July, August, and September) in the facility. Randomisation was done using a Stata program using the runiform function. The randomisation was as balanced as possible within districts and facility type.

Three training workshops will be conducted for health care providers from each participating facility, before commencing the first audit cycle. There will be three workshops, one for health care providers in each stratum. During these workshops staff will be trained in the conduct of standard based audits. During the workshop they will also identify standards of most relevance to their facility which can be audited. Staff from each CEmOC facility will be asked to select 6 such standards for which the facility usually has at least 25 clients per month. Staff from BEmOC facilities will be asked to select 4 standards. They will rank these standards in terms of their importance to the facility.

The trial statistician will then assign standards to be audited within each facility in a manner which assigns the highest-ranking standards for each facility subject to the number in the stratum not exceeding six facilities for any given standard. In determining the standards to be audited at each facility priority will be given to the more popular standards, to maximise the number of individual standards for which there are close to 18 facilities auditing the standard following a balanced incomplete stepped wedge design.

The sequence in which standards are audited within each facility will be assigned using a Stata program using the runiform() function to randomise the facilities to quarters (1 or 2) for each standard. The allocation will be constrained to ensure that for each facility the appropriate number of standards are assigned to each quarter.

**Data Collection**

As indicated in Figure 1 each of the standards to be audited at a facility will be assessed during the first and third month of the audit cycle for that standard. In addition, it will also be assessed during the first month of the first audit cycle at the facility (if the audit cycle is in the second quarter) or during the last month of the second quarter (if the audit cycle is in the first quarter) but not during other study months. Tables 2 and 3 illustrate the assessments to be performed for standards audited within a CEmOC facility in stratum which is assigned to audit for standards denoted as standards A, B, C and D, and a BEmOC facility in stratum II which is assigned to audit for standards denoted as standards E and F.

**Table 2: Timing of assessments for a CEmOC facility in Stratum I at which standards A, B, C and D are audited**

| Representation | Standard | Quarter 1 | | | Quarter 2 | | |
| --- | --- | --- | --- | --- | --- | --- | --- |
|  |  | July | Aug | Sept | Oct | Nov | Dec |
| By standard | A | X(a) |  | X(a) |  |  | X(a) |
|  | B | X(b) |  |  | X(b) |  | X(b) |
|  | C | X(c) |  | X(c) |  |  | X(c) |
|  | D | X(d) |  |  | X(d) |  | X(d) |
|  |  |  |  |  |  |  |  |
| As a facility | A, B, C and D | X(a,b,c,d) | A | X(a,c) | X(b,d) | B | X(a,b,c,d) |

Grey shaded boxes represent action being taken to address the standard being considered in that quarter. X(a) indicates that data for Standard A will be collected in that month, X(b), X(c) and X(d) are similarly defined

X(a,b,c,d) indicates that data for Standards A, B, C and D will be collected in that month

**Table 3: Timing of assessments for a BEmOC facility in Stratum II at which standards E and F are audited**

| Standard | Quarter 1 | | | Quarter 2 | | |
| --- | --- | --- | --- | --- | --- | --- |
|  | Aug | Sept | Oct | Nov | Dec | Jan |
| E | X(e) |  | X(e) |  |  | X(e) |
| F | X(f) |  |  | X(f) |  | X(f) |

Grey shaded boxes represent action being taken to address the standard being considered in that quarter. X(e) indicates that data for Standard E will be collected in that month, X(f) is similarly defined

Data for each standard will be collected by staff within the facility using a standard data collection tool, to obtain data for 25 clients within the relevant month either by conducting an exit interview or by extracting data from registers, case notes or partographs within facilities. When the number of eligible participants in the month is not expected to exceed 50 a consecutive sample of eligible clients within the facility will be obtained until the required number of 25 is reached. When more than 50 clients are anticipated systematic random sampling will be used to obtain the sample.

**Eligibility**

*Participant inclusion criteria*

- Clusters: 43 healthcare facilities providing emergency obstetric and newborn care within five districts in Malawi.
- Clients: women and their newborns who attend the study facilities for obstetric / newborn care addressed by the standard being assessed that month

**Participant type**

- Women and / or their newborns receiving obstetric or newborn care

**Target number of participants**

For each standard audited at a facility it is anticipated that care received by 25 clients will be assessed in each of three study months (as illustrated in Figure 1 and Tables 2 and 3). At each of the ten participating CEmOC designated facilities four standards are to be audited, thus the total clients participating at each CEmOC is expected to be 300. At each of the thirty-three participating BEmOC designated facilities two standards are to be audited, thus the total clients participating at each BEmOC is expected to be 150. The total number of participants is thus expected to be 8,100.

**Sample size considerations**

For sample size calculations various parameters need to be specified. These include the intracluster correlation (ICC) and the cluster autocorrelation (CAC). The ICC refers to the correlation between values from individuals in the same cluster on the same occasion. The CAC measures the correlation between means within the same cluster at different times. It is assumed that data for each standard are collected according to the incomplete stepped wedge design shown in Figure 1, with three clusters following each sequence in the design. Sample size calculations were performed to determine differences detectable using the Rshiny app which was developed by Karla Hemming, a Professor of Biostatistics whose research is focused on the design and analysis of stepped wedge trials (available at: <https://clusterrcts.shinyapps.io/rshinyapp/>).

Table 4 indicates the percentage change in compliance for the standard that is detectable for a single standard, with either 80% or 90% power, an ICC of either 0.1 or 0.2, and a CAC of 0.8 or 0.5 using a sample of 25 clients for each assessment month in each cluster. These calculations focused on the proportion of respondents who deemed the standard to have been achieved as a binary response, assuming that the initial compliance level for the standard is 50%.

**Table 4 Differences detectable (percentage) for using an incomplete stepped wedge design with specified number of facilities conducting a standards-based audit in a balanced incomplete stepped wedge design**

| **Sample size for each facility-month** | **Number of facilities doing standards-based audit** | **ICC** | **CAC** | **Power** | |
| --- | --- | --- | --- | --- | --- |
|  |  |  |  | **80%** | **90%** |
| 25 | 12 | 0.1 | 0.5 | 21.1% | 24.0% |
|  |  |  | 0.8 | 18.3% | 20.9% |
|  |  | 0.2 | 0.5 | 26.0% | 29.3% |
|  |  |  | 0.8 | 20.9% | 23.9% |
| 25 | 18 | 0.1 | 0.5 | 17.5% | 20.0% |
|  |  |  | 0.8 | 15.2% | 17.5% |
|  |  | 0.2 | 0.5 | 21.6% | 24.7% |
|  |  |  | 0.8 | 17.5% | 20.0% |
| 10 | 18 | 0.1 | 0.5 | 21.7% | 24.7% |
|  |  |  | 0.8 | 20.5% | 23.5% |
|  |  | 0.2 | 0.5 | 25.6% | 28.9% |
|  |  |  | 0.8 | 23.0% | 26.1% |

These calculations indicate that there will be at least 80% power to detect an improvement in compliance of 22% provided the ICC does not exceed 0.2 and the CAC does not fall below 0.5. If the initial compliance level is lower or higher than 50% then the difference detectable will be smaller. For standards that are audited in fewer clients per cluster or in fewer clusters the difference detectable will be larger. To indicate the impact of each of these reductions in sample size the differences detectable are also provided for 10 clients per cluster with 18 facilities and for 25 clients per cluster with 12 facilities auditing the standard.

**Statistical Analysis**

Each standard considered will be analysed separately to provide an estimate of the impact of the audit of that standard on compliance with the standard. Analysis will use the binary responses of respondents within facilities in multilevel mixed effects logistic regression models, with fixed effects for intervention and facility type, and random effects for month, facility (cluster) and month by facility (cluster) interaction. District will also be considered for inclusion as a random effect. For each standard the impact of the intervention will be estimated with a 95% confidence interval. Compliance with defined standard of care, aggregated for all standards audited will also be estimated in a similar manner, with a 95% confidence interval.

**Ethical approval**

Ethics committee of Liverpool School of Tropical Medicine Research Protocol (18-028)2.

Study granted ethics review exemption by the Malawi Ministry of Health, 20/06/2018 (Ref: QMD/10)

**Dissemination**

A dissemination meeting will be planned at the end of the study where k Key Ministry of health Officials and district officers and health care providers from target district and facilities will be invited to attend. Publication of peer review papers – the aim is to publish 1 paper.

**References**

1. World Health Organization, UNICEF, UNFPA, the World Bank, the United Nations Population Division. Trends in Maternal Mortality to 2015: Estimates by the WHO, UNICEF, UNFPA. Trends in Maternal Mortality 1990 to 2015: Estimates by the WHO, UNICEF, UNFPA, The World Bank and the United Nations Population Division. Geneva
2. United Nations Children’s Fund, World Health Organization, World Bank Group UNICE. The United Nations Population Division 2017. Levels & Trends in child mortality. Estimates Developed by the UN Inter-agency Group for Child Mortality Estimation. Available on <https://www.unicef.org/publications/files/Child_Mortality_Report_2017.pdf>. Accede on 19^th^ July 2108

Jamtvedt G, Young JM, Kristoffersen DT, O’Brien MA, Oxman AD. Audit and feedback: effects on professional practice and healthcare outcomes. Cochrane Database Syst Rev 2012;(6):CD000259.

1. Kongnyuy EJ, Mlava G, van den Broek N. Criteria-based audit to improve a district referral system in Malawi: a pilot study. BMC Health Serv Res 2008;8:190.
2. Kidanto H, Mogren I, Massawe S et al. Criteria-based audit on management of eclampsia patients at a tertiary hospital in Dar es Salaam, Tanzania. BMC Pregnancy Childbirth 2009;9:13.

Pirkle CM, Dumont A, Traore M, Zunzunegui M-V. Validity and reliability of criterion based clinical audit to assess obstetrical quality of care in West Africa. BMC Pregnancy and Childbirth. 2012. p. 118

Kidanto HL, Wangwe P, Kilewo CD, Nystrom L, Lindmark G. Improved quality of management of eclampsia patients through criteria based audit at Muhimbili National Hospital, Dar es Salaam, Tanzania. Bridging the quality gap. BMC Pregnancy Childbirth. 2012; 12: 134. doi: 10.1186/1471-2393-12- 134 PMID: 23170817

1. World Health Organisation. Beyond the numbers: reviewing maternal deaths and complications to make pregnancy safer. www.who.int. http://www.who.int/making_pregnancy_safer/documents/9241591838/en/index.html. Published 2004.

World Health Organisation; Standards for Maternal and Neonatal Care? Available on <http://www.who.int/reproductivehealth/publications/maternal_perinatal_health/intro.pdf>. Accessed on 12 July 2018.

1. Graham WJ. Criterion-based clinical audit in obstetrics: bridging the quality gap. Best Pract Res Clin Obstet Gynaecol 2009;23:375–88.

Malawi Demographic Health Survey 2015-2016. National Statistics Malawi

1. United Nations. Sustainable development goals. 2015. http://www.un.org/Sustainable development/sustainable-development-goals/. Accessed 10 July 2018. 2015

Unicef, WHO. Every newborn: an action plan to end preventable deaths. 2014. Available on www.who.int/maternal_child_ adolescent/topics/newborn/enap_consultation/en/. Accessed on 10^th^ June 2018

WHO. Strategies toward ending preventable maternal mortality (EPMM). 2015. Available on http://who.int/reproductivehealth/ topics/maternal_perinatal/epmm/en/. Accessed on 10^th^ June 2018

World health organisation. Global Programme on Evidence for Health Policy World Health Organization Geneva 2003, Switzerland aavailable on http://whqlibdoc.who.int/hq/2003/EIP_GPE_EQC_2003_1.pdf ). Accessed on 1^st^ August 2018

**Appendix 1: Examples of General Standards of Care and Standards for Obstetric & Newborn Complications**

| 1. General Standards of Care | |
| --- | --- |
| 1.1 | Every woman seeking care for a complication during or after pregnancy is attended to by a skilled healthcare provider within 30 minutes after arrival at the healthcare facility |
| 1.2* | All women attending intrapartum care are received and treated with respect |
| 1.3* | All women who give birth at a healthcare facility are given the opportunity to have a companion of choice |

| 1. Management of Hemorrhage | |
| --- | --- |
| 2.1* | As part of active management of the third stage of labour, all women give birth at the healthcare facility receive an oxytocic. |
| 2.2 | Every woman with a retained placenta undergoes manual removal of the placenta within 1 hour of the diagnosis |
| 2.3 | All women who have an antepartum (APH) or postpartum haemorrhage (PPH) have an IV line inserted within 15 minutes of the diagnosis |
| 2.4* | All women who have a APH or PPH have their haemoglobin (Hb) checked and recorded |

| 1. Management of Infection and Sepsis | |
| --- | --- |
| 3.1* | All women who have an uncomplicated birth at the healthcare facility have their temperature measured and recorded at least once after birth and before discharge |
| 3.2 | All women with fever are tested for malaria within 24 hours |
| 3.3 | Women with a suspected diagnosis of infection or sepsis are started on antibiotic treatment within one hour of the diagnosis being made |
| 3.4 | All women who require caesarean section are given prophylactic antibiotics |

| 1. Management of (Pre-) Eclampsia | |
| --- | --- |
| 4.1* | Every woman in labour has her blood pressure measured, urine tested for protein and the results recorded |
| 4.2 | Women with high blood pressure during labour or birth are given anti-hypertensive treatment |
| 4.3 | Every woman who has an eclamptic fit is given magnesium sulphate or diazepam IV or IM within 5 minutes of the fit occurring |
| 4.4 | Every woman with pre-eclampsia or eclampsia has a fluid input-output chart completed |
| 4.5 | All mothers attending ANC have their BP checked and urine tested for protein |

| 1. Management of Prolonged and Obstructed Labour | |
| --- | --- |
| 5.1* | Every woman in labour in a healthcare facility is monitored using a partograph correctly |
| 5.2 | All women who need an emergency Caesarean section should be delivered within 60 minutes of the decision |

| 1. Management of Abortion | |
| --- | --- |
| 6.1 | Every woman with an incomplete abortion undergoes evacuation / manual vacuum aspiration within 24 hours of arrival at the healthcare facility |
| 6.2 | Every woman who had a miscarriage (complete or incomplete) is provided with advice on contraception before discharge home |
| 6.3 | Every woman who has had uterine evacuation or Manual Vacuum Aspiration (MVA) has a clinical examination before being discharged home |
| 6.4 | Every woman who has had uterine evacuation or MVA has her temperature measured and recorded before she is discharged home |

| 1. Newborn Care | |
| --- | --- |
| 7.1 | Every newborn baby delivered in the facility is weighed after birth |
| 7.2 | All newborn babies with temperature of 38^0^ C or more should be started on antibiotics within 12 hours of the measurement |
| 7.3 | Low Birth Weight (LBW) babies receive Kangaroo Mother Care (KMC) |

*Indicates a Tracer Standard

**Appendix 2: UNICEF health facilities by districts and level of health care**

| **District** | **No** | **Facility** | **Level** |
| --- | --- | --- | --- |
| Dedza | 1 | Dedza District hospital | CEmOC |
|  | 2 | Mua mission hospital | CEmOC |
|  | 3 | Mtakataka | BEmOC |
|  | 4 | Golomoti | BEmOC |
|  | 5 | Kaphuka | BEmOC |
|  | 6 | Mayani | BEmOC |
|  | 7 | Kasina | BEmOC |
|  | 8 | Chitowo | BEmOC |
|  | 9 | Lobi | BEmOC |
|  | 10 | Chimoto | BEmOC |
|  | 11 | Chikuse | BEmOC |
|  | 12 | Mtendere | BEmOC |
| Blantyre |  |  |  |
|  | 1 | QECH | CEmOC |
|  | 2 | Mlambe hospital | CEmOC |
|  | 3 | Bangwe | BEmOC |
|  | 4 | Chavala | BEmOC |
|  | 5 | Chikowa | BEmOC |
|  | 6 | Chileka | BEmOC |
|  | 7 | Chileka SDA | BEmOC |
|  | 8 | Chilomoni | BEmOC |
|  | 9 | Chmembe | BEmOC |
|  | 10 | Chirimba | Dispensary |
|  | 11 | Dziwe | BEmOC |
|  | 12 | Gate way | Dispensary |
|  | 13 | Kadidi | Dispensary |
|  | 14 | Limbe | BEmOC |
|  | 15 | Lirangwe | BEmOC |
|  | 16 | Lundu | BEmOC |
|  | 17 | Madziabango | BEmOC |
|  | 18 | Makata | Dispensary |
|  | 19 | Makhetha | Dispensary |
|  | 20 | Malabada | BEmOC |
|  | 21 | Mdeka | BEmOC |
|  | 22 | Mlambe | BEmOC |
|  | 23 | Mpemba | BEmOC |
|  | 24 | Namikoko | Dispensary |
|  | 25 | Ndirande | BEmOC to be CEmOC |
|  | 26 | Saint Vinicent | BEmOC |
|  | 27 | Soche maternity | Dispensary |
|  | 28 | South Lunzu | BEmOC |
|  | 29 | Zingwangwa | BEmOC |
|  | 30 | Pensulo | BEmOC |
| Mangochi |  |  |  |
|  | 1 | Mangochi D Hospital | CEmOC |
|  | 2 | Mulibwanji | CEmOC |
|  | 3 | Monkeybay | CEmOC |
|  | 4 | St Martins | CEmOC |
|  | 5 | Kapire | Non BEmOC |
|  | 6 | Chilipa | BEmOC |
|  | 7 | Phirilongwe | BEmOC |
|  | 8 | Mtimabii | Targeted BEmOC |
|  | 9 | Katema | Non BEmOC |
|  | 10 | Chlilonga | BEmOC |
|  | 11 | Nankumba | BEmOC |
|  | 12 | Malembo | Non BEmOC |
|  | 13 | Nankhwali | Non BEmOC |
|  | 14 | Nkope | Targeted BEmOC |
|  | 15 | Koche | BEmOC |
|  | 16 | Lungwena | BEmOC |
|  | 17 | Namalaka | Non BEmOC |
|  | 18 | Lugola | Non BEmOC |
|  | 19 | Makanjira | BEmOC |
|  | 20 | Lulanga | Non BEmOC |
|  | 21 | Malukula | Non BEmOC |
|  | 22 | Mase | Non BEmOC |
|  | 23 | Chikole | Non BEmOC |
|  | 24 | Katuli | Targeted BEmOC |
|  | 25 | Ngapani | Non BEmOC |
|  | 26 | Luwalika | Non BEmOC |
|  | 27 | Jalasi | Non BEmOC |
|  | 28 | Namwera | BEmOC |
|  | 29 | Sr Martha | Non BEmOC |
|  | 30 | Nangalamu | Non BEmOC |
|  | 31 | Mkumba | BEmOC |
|  | 32 | Mpondasi | Non BEmOC |
| Thyolo |  |  |  |
|  | 1 | Thyolo D hospital | CEmOC |
|  | 2 | Malamulo hospital | CEmOC |
|  | 3 | Chisoka | BEmOC |
|  | 4 | Bvumbwe | BEmOC |
|  | 5 | Mikolongwe | BEmOC |
|  | 6 | Chimaliro | BEmOC |
|  | 7 | Khonjeni | BEmOC |
|  | 8 | Thekerani | BEmOC |
|  | 9 | Mangunda | Non BEmOC |
|  | 10 | Gombe | Non BEmOC |
|  | 11 | Mapanga | Non BEmOC |
|  | 12 | Chipho | Non BEmOC |
|  | 13 | Changata | Non BEmOC |
|  | 14 | Molere | Non BEmOC |
|  | 15 | Mianga | Non BEmOC |
|  | 16 | Didi | Non BEmOC |
|  | 17 | Thomasi | Non BEmOC |
|  | 18 | Makungwa | Non BEmOC |
|  | 19 | Chimvu | Non BEmOC |
|  | 20 | Chingazi | Non BEmOC |
|  | 21 | Mitengo | Non BEmOC |
|  | 22 | Makapwa | Non BEmOC |
|  | 23 | Nchima | Non BEmOC |
|  | 24 | Makandi | Non BEmOC |
|  | 25 | Satemwa | Non BEmOC |
|  | 26 | Sambankhanja | Non BEmOC |
|  | 27 | Chimaliro | BEmOC |
| Nkhatabay |  |  |  |
|  | 1 | Nkhatabay D hospital |  |
|  | 2 | Bula |  |
|  | 3 | Chesamu |  |
|  | 4 | Chikwina | BEmOC |
|  | 5 | Chilambwe |  |
|  | 6 | Chintheche | CEmOC |
|  | 7 | Chisala | BEmOC |
|  | 8 | Chitheka | BEmOC |
|  | 9 | Kachere | BEmOC |
|  | 10 | Kande | BEmOC |
|  | 11 | Kavuzi |  |
|  | 12 | Khondowe |  |
|  | 13 | Liuzi |  |
|  | 14 | Lwazi |  |
|  | 15 | Maula |  |
|  | 16 | Mpamba | BEmOC |
|  | 17 | Mzenga |  |
|  | 18 | Nthungwa | BEmOC |
|  | 19 | Usisya | BEmOC |
