# Supplementary material for: Standards-based audit to improve quality of maternal and newborn care—A stepped-wedge cluster randomised trial in Malawi
Source: PLoS One. 2024 Sep 30;19(9):e0310896. doi: 10.1371/journal.pone.0310896 (PMC11441693; doi:10.1371/journal.pone.0310896)
Supplement: Attachment — Submitted filename: Responses to Reviewers.pdf [file pone.0310896.s012.pdf]

Response to reviewers

Manuscript: Standards-based audit to improve quality of maternal and newborn care – a stepped-wedge cluster randomised trial in Malawi (PONE-D-23-41107).

Comment	Response
Reviewer 1	
1- Line 312: Provide a measure of dispersion for the mean of 20.6.	Thank you. This has been added.
2- Table 1: Define all abbreviations.	Thank you. This has now been done
3- Indicate if an effect for the repeated measures from individual patients was included in the logistic regression. These correlated measures need to be accounted for in the models.	Please note that there was no intention to measure any individual client multiple times, so no identification details were captured as indicated on lines 212-3. Therefore patient was not included in the model<br>Clusters (healthcare facilities) were however assessed repeatedly for each standard assessed and therefore models fitted did include effects for cluster and for interactions of cluster with Month (line 236) and with Standard (lines 386-9)
4- Line 378: The common abbreviation for standard deviation is SD.	Thank you this has been amended
5- Cite the statistical software used for the analysis.	This has been added in line 229
Reviewer 2	
Areas for improvement<br>Title:<br>The authors should write specific and concise title reflecting the study. I think no need to mention cluster randomized trial, they can add cross sectional study.	Thank you for your suggestion. We have checked the requirements again:<br>The title has 17 words (102 characters) which is within the requirements.<br>CONSORT guidelines indicate that the design should be indicated in the title. Since the design is cluster randomized, we have retained this term in the title.
Abstract:<br>The authors should revise the language to improve readability.<br>The authors should select appropriate key words.<br>The authors should make sure that the abstract don't exceed 300 words.<br>The authors should use IMRaD format to write the abstract.	Thank you for your suggestions.<br>We have revised the Abstract as best as possible within the word limit permitted (300 and the abstract is 296 words).<br>IMRaD headings have been added and the format used by PLosOne has been followed.
The authors should revise introduction section for grammar issues and language to improve readability.	We have revised the Introduction and have again checked all English grammar and spelling throughout the manuscript.
The authors used statistics in first paragraph of introduction section, they should mention from where this statistics came, which mean from which country.	As stated in the opening sentence these statistics are global statistics as this seems most relevant in this manuscript and the Introduction.

The authors should join some related paragraphs together.	Three paragraphs have been joined (now lines 93 - 107)
The authors should mention the gap clearly to the reader and the need for the study.	The gap (between availability and quality) is mentioned in the introduction (now 120-22).
Study design, the authors should mention clearly the designed used and the important of the study design.	The study design section (lines 129-138) identifies the trial design used and explains the reason this design was selected
Study setting, The authors should revise the language to improve readability.	We have re-checked this section and revised the text to improve the language.
Training on standards based audit, The authors should revise the language to improve readability.	We have re-checked this section and revised the text to improve the language.
Assigning standards, the authors should revise the language to improve readability and complete the missing information.	We have re-checked this section including language and clarified it further.
Data collection, the authors should mention if they used any tool to collect data. For the tool used in this study, they authors should mention from where they obtained this tool? Is it adopted or developed by the authors. If developed by authors how they check the validity and reliability of the tool?	The data collection section explains that standard-specific tools were developed for the study (lines 206-8).<br>The wording has been revised to indicate more clearly that the tools used were developed specifically for each standard and for the study. Each tool captured data regarding the criteria to be fulfilled for there to be compliance with the relevant standard.
Data analysis, The authors should revise the langue to improve readability.	We have re-checked this section and clarified the language used.
Results:<br>The authors should follow appropriate labelling of the title of each table in the manuscripts according to the journal's guidelines. While the study appears to be sound, the language is unclear, making difficult to follow. Thus, the authors should work with a writing editor to improve readability of the text.	Table titles have been revised to improve clarity of content and ensure they are brief and descriptive<br><br>We have carefully reviewed the text, made some changes to clarify details and hope we have now met your expectations of readability for a global audience.
References:<br>The authors should revise all references according to the guidelines provided.	We believe we have provided the references in line with the instructions for the journal, but did find a couple of errors which have now been corrected.
Reviewer 3	
major revision.<br>- Can the description of Malawi level of healthcare delivery be included in the introduction? Emergency obstetric care take place at the secondary and tertiary level. The write up did not tell us at which level.	Thank you for these comments.<br>We have included 'levels' in the Introduction section (hospital – secondary level and health centre – primary level) for Comprehensive and Basic EmONC respectively.<br>In Malawi hospitals are generally considered secondary level and health centres are primary level. We have clarified this in the manuscript in the Methods section (setting)

- The analysis also did not tell us what the adherence to this standard was at these two levels.	Many thanks for this suggestion – we have now added statistics for both levels in the Results section and the Abstract
- The introduction also did not tell us whether both private and public facilities were used. Mission hospital was later mentioned (Line 290). This shows a mixed of the hospital types.	There is no active private sector providing EmONC (either basic or comprehensive) in any of the 5 districts included in the study.<br>'Mission' hospitals provide public healthcare in the same was as hospitals that are run only by the Ministry of Health in Malawi.<br>We have amended the text to avoid any confusion. The main difference in the health care facility types was whether they were a hospital or a health centre and whether they were providing Basic or Comprehensive EmONC. We have provided these details in Supplementary Table 2.<br><br>Thanks for this comment.
- How does the standard relate to the WHO quality of care (QoC) standards? Were any indicators adopted from the WHO QoC measurement?	Thank you for this question.<br>The WHO standards were used as reference but are not formatted to allow for audit.
- The table illustrating the 25 standards should have been added to the main document.	All 25 standards are provided in the Supplementary Material. (Supplementary Table 3)<br>The 12 standards which were subjected to audit are listed in Table 1.<br>The other 13 standards were not used so we do not think they need to be moved from the supplementary material into the manuscript as this would make the manuscript overly long.
- The standard should have been categorized with relatable descriptive terms. This would have made the analysis easy to read and relate to.	Thank you for this feedback, standards are now referred to using terms that help identify them rather than numbers
- Under result, mentioning the standard that health facilities audited would have made the segment clear. (Line 269 -268). Line 362 -363 should be moved to the beginning of the analysis. It has basic information that would make the analysis clear.	Information on the standards audited at each facility is provided in Supplementary Table 4.<br>Standards are now referred to by description rather than number.<br>As explained under Trial outcomes (lines 150-1) the specific standards audited were not of interest.
- Line 309 mentioned all 12 standards. This contradict the 25 standard that was mentioned in line 29 -20.	There were 25 standards available from which to choose. Only 12 standards were actually audited; we have inserted 'used' on line 316 to clarify this
- Line 415 – 434. The discussion did not adequately explain the analysis	We have amended the text as best as we can hopefully clarifying all points raised.<br>Many thanks for all the comments and critical reading of the manuscript.
- Line 427 -429 (The statement is not clear)	Thank you for highlighting this - we agree that the statement could be clarified and have added further detail both in the discussion and the relevant results section.

the author should review and rewrite the analysis and discussion.	We have amended the text as best as we can hopefully clarifying all points raised.<br>Many thanks for all the comments and critical reading of the manuscript.
Editors comments	
1 Please ensure that your manuscript meets PLOS ONE's style requirements	Thank you. The heading styles have been updated to comply with your requirements.
2 Please state the name of the registry and the registration number . . . on the title page of your manuscript	These details were included in submission data and are now included on the title page of the manuscript.
3 Please state what role the funders took in the study.	The statement suggested is correct and is included in the cover letter.
4 Please include captions for your Supporting Information files at the end of your manuscript, and update any in-text citations to match accordingly.	The supporting information has been restructured to comply with your requirements. The tracked changed manuscript contains the changes made to supplementary information but the clean version only contains the necessary captions. (Insertion of the supplementary material document was not tracked, but subsequent edits were).
5 Please review your reference list to ensure that it is complete and correct.	These have been checked
